# Cardiovascular disease risk prediction models in the Chinese population- a systematic review and meta-analysis

**DOI:** 10.1186/s12889-022-13995-z

**Published:** 2022-08-24

**Authors:** Guo Zhiting, Tang Jiaying, Han Haiying, Zhang Yuping, Yu Qunfei, Jin Jingfen

**Affiliations:** 1 Nursing Department, The Second Affiliated Hospital of Zhejiang University School of Medicine (SAHZU), No.88 Jiefang road, Shangcheng District, Hangzhou, 310009 Zhejiang Province China; 2 Zhejiang University City College, No. 51 Huzhou Street, Gongshu District, Hangzhou, 310015 Zhejiang Province China; 3 Key Laboratory of The Diagnosis and Treatment of Severe Trauma and Burn of Zhejiang Province, Hangzhou, 310009 Zhejiang Province China

**Keywords:** Cardiovascular diseases, Prediction model, Risk

## Abstract

**Background:**

There is an increasing prevalence of cardiovascular disease (CVD) in China, which represents the leading cause of mortality. Precise CVD risk identification is the fundamental prevention component. This study sought to systematically review the CVD risk prediction models derived and/or validated in the Chinese population to promote primary CVD prevention.

**Methods:**

Reports were included if they derived or validated one or more CVD risk prediction models in the Chinese population. PubMed, Embase, CINAHL, Web of Science, Scopus, China National Knowledge Infrastructure (CNKI), VIP database*,* etc.*,* were searched. The risk of bias was assessed with the Prediction Model Risk of Bias Assessment Tool (PROBAST). Meta-analysis was performed in R using the package metamisc.

**Results:**

From 55,183 records, 22 studies were included. Twelve studies derived 18 CVD risk prediction models, of which seven models were derived based on a multicentre cohort including more than two provinces of mainland China, and one was a model developed based on a New Zealand cohort including Chinese individuals. The number of predictors ranged from 6 to 22. The definitions of predicted outcomes showed considerable heterogeneity. Fourteen articles described 29 validations of 8 models. The Framingham model and pooled cohort equations (PCEs) are the most frequently validated foreign tools. Discrimination was acceptable and similar for men and women among models (0.60–0.83). The calibration estimates changed substantially from one population to another. Prediction for atherosclerotic cardiovascular disease Risk in China (China-PAR) showed good calibration [observed/expected events ratio = 0.99, *95% PI* (0.57,1.70)] and female sex [1.10, *95% PI* (0.23,5.16)].

**Conclusions:**

Several models have been developed or validated in the Chinese population. The usefulness of most of the models remains unclear due to incomplete external validation and head-to-head comparison. Future research should focus on externally validating or tailoring these models to local settings.

**Trail registration:**

This systematic review was registered at PROSPERO (International Prospective Register of Systematic Reviews, CRD42021277453).

**Supplementary Information:**

The online version contains supplementary material available at 10.1186/s12889-022-13995-z.

## Background

Cardiovascular disease (CVD) is the leading cause of mortality and a major contributor to disability worldwide, which led to 18.6 million deaths in 2019 [1]. Internationally, China and India have the highest burdens of CVD [2]. There is an increasing prevalence of CVD in China, where it represents the leading cause of mortality [3, 4].

In an attempt to mitigate the risk and reduce the burden of cardiovascular disease in such a vast country, implementing an overall risk-based prevention approach has been confirmed as a cost-effective method [5, 6]. However, it is critical to know that precise CVD risk identification is the fundamental prevention component [7]. Inappropriate risk-based CVD management may lead to undertreatment or overtreatment. Risk assessment offers a platform for communication between health care providers and patients, improving patients’ perception of risk and promoting shared decision-making, which ultimately enhances patients’ adherence to medical treatment and health lifestyle modification [8].

Over the past two decades, more than 360 CVD risk prediction models have been developed through one or several longitudinal cohorts since the pioneering Framingham research [9]. However, these equations were mostly derived from Caucasian populations, and population ethnicity and region have roles in modifying cardiovascular risk [10]. As a result, these models cannot be used interchangeably without recalibration because of the different risk factor profiles (i.e., lower level of total cholesterol and higher absolute hypertension burden in China) and CVD profiles (i.e., higher stroke/cardiovascular disease ratio in China) between western and Chinese populations [8]. The Framingham risk equations and American College of Cardiology/American Heart Association Pooled Cohorts Equations (PCEs) were most commonly validated in the Chinese population, with a broad range overestimation in men and underestimation in women [11–14]. Several CVD risk prediction models have been developed based on the large sample size of Chinese adults since 2003, such as the 10-year risk prediction model of CVD in Chinese [15–17], Prediction for atherosclerotic cardiovascular disease Risk in China (China-PAR) [13], and the 5-year risk prediction model of CVD [18]. However, it is not clear which is currently the most appropriate tool for Chinese cardiovascular disease risk prediction.

The aim of this study was to systematically review the published research that derived or validated one or more CVD risk prediction models in China, followed by a formal meta-analysis to summarize and compare the overall predictive performance of these models to inform the choice of the risk model in China.

## Methods

This systematic review protocol was registered at PROSPERO (International Prospective Register of Systematic Reviews) (CRD 42021277453). We followed the PRISMA (Preferred Reporting Items for Systematic Reviews and Meta-analyses) guidelines published by the Cochrane Prognosis Methods Group [19], and the checklist for systematic reviews and meta-analyses of prediction modelling studies [20] was used to conduct our systematic review.

### Literature search

For this review, we used the search details of the review by *Damen* et al. [9] on all CVD prediction models for the general population. In the original search in 2013, two databases (Medline and Embase) were searched. As shown by this review, several CVD prediction models have been developed in recent years. Therefore, we complemented the search details by updating their search and expanding the search database. This search strategy was translated appropriately for Embase, CINAHL, Web of Science (Core Collection), and Scopus. We also developed a Chinese search strategy combining subject indexing terms and free-text search terms in the title and abstract fields in the Chinese Biomedical Literature Service System (SinoMed). This search strategy was translated appropriately for the Wan Fang Database, China National Knowledge Infrastructure (CNKI) and VIP database. A search of Open Grey (OpenGrey, 2019) and Google Scholar was conducted to obtain potential grey literature. The search strategy is presented in Table S1. We systematically searched electronic databases from inception to September 10, 2021. This systematic review was limited to studies conducted in humans and published in English or Chinese. For external validation studies where the development study was not screened by our search, we manually retrieved the original article through citation.

### Eligibility criteria

We included all primary articles that reported one or more multivariable prediction models or scores that have been suggested for individual risk estimation of any future CVD outcome. However, in the full-text screening, we included only models developed or validated in the Chinese population. We defined ‘model developed in Chinese population’ as 1) the risk models specifically developed to predict CVD in Chinese and 2) the original cohort of model construction including Chinese; moreover, this model can be used in Chinese population CVD risk estimation. For the validation papers, both studies that validated prediction models only in the Chinese population and published validation data containing Chinese were included. The type of model presentation was not limited. Additionally, two or more presented model types yielding the same predictor-outcome associations with some baseline hazard or risk estimate were considered as one model.

We excluded articles without a defined end-point and describing models for predicting the risk of venous disease or stroke alone; validation articles with a cross-sectional study design that, for instance, compared predicted risks of two different models at one time point without any association with actual CVD outcomes; carotid endarterial plaque detected by carotid ultrasound was used as a surrogate of CVD end point event in the studies; studies reporting on the incremental value of one or more new predictors to existing models [9] and investigating a single predictor [21]; and articles describing models developed from or validated exclusively in specific populations, such as patients with diabetes, with HIV, with atrial fibrillation, or undergoing any cardiac surgery.

### Screening process

Initially, pairs of two trained reviewers (Guo ZT and Tang JY) independently screened retrieved records for eligibility on the title and subsequently on the abstract. Then, the full text of the remaining studies was obtained. The same two reviewers examined potentially relevant studies according to the predetermined eligibility criteria. Disagreements were resolved by discussion with an advisor (Zhang YP).

### Data extraction

The eligible articles were categorized into two groups: development articles and external validation articles. A standardized electronic form followed by the checklist for appraisal and data extraction for systematic reviews of prediction modelling studies (CHARMS checklist) [22] was constructed to facilitate the data extraction process (Table S2). Information extracted from studies describing model development included study design, location, cohort information, prediction horizon, predicted outcome, predictors, modelling method, method of internal validation, the number of study participants and CVD events, model presentation (e.g., regression equation or risk chart) and predictive performance.

For studies describing external validation of a prediction model, we extracted the study design, prediction model, cohort information, predicted outcome(s), prediction horizon, the number of study participants, observed and expected CVD events, and the model’s predictive performance before model recalibration. If an article described multiple models or validated them in different cohorts, we carried out separate data extraction for each model or cohort. We sought clarification from the authors using email communication if important information was missing. In addition, for prediction models with a time-to-event outcome, it is important to note that the extracted values for observed events should be based on Kaplan–Meier estimates [23].

One reviewer (Guo ZT) screened the full-text articles and extracted data from the included studies. A second reviewer (Han HY) checked the exact items. For any disagreements, a third (Zhang YP) reviewer was involved in reaching a consensus.

### Critical appraisal

We adopted PROBAST to assess the risk of bias from 4 aspects: participants, predictors, outcome and analysis, which can cause distorted estimation of a prediction model’s performance; in addition, PROBAST can also evaluate the applicability of a prediction model [22, 23]. The signalling questions were answered as yes, probably yes, probably no, no and no information. The results of risk of bias had 3 potential outcomes: low, high or unclear risk of bias. A positive answer suggests no risk of bias. Two reviewers (Tang JY, Yu QF) independently assessed the methodological quality (risk of bias) and applicability of the included studies. If there were any disagreements, they were resolved by discussion and consultation with an advisor (Jin JF) to reach a consensus.

### Reliability and clinical usability of available models

Reliability was defined using the following criteria: 1) models validated externally in a separate investigation/paper, 2) C statistic > 0.70 [24], and 3) overestimated/underestimate rate lower than 100% [25]. For reliable models, clinical usability was assessed by 4 items: 1) 10 predictors or fewer, 2) no more than one medical resource needed, 3) full equation or risk chart reported and 4) availability of an online calculator [24, 26]. All eligible models included were evaluated. The mean C statistic and summarized overestimated/underestimate rate were used when more than one validation was reported.

### Statistical analysis

The pooled c-statistic and OE ratio was performed by meta-analysis for each prediction model. For those articles that did not report the OE ratio, we calculated or estimated it through other data listed in the papers using the equations recommended by Debray et al. [20] Additionally, some studies validated cohorts shorter than 10 years in which these models were initially designed to predict 10-year CVD events; we extrapolated observed event risk (*P*_*O*_) and expected event risk (*P*_*E*_) separately to 10 years using the equation based on Poisson distribution, and observed 10-year cumulative events were calculated through Kaplan–Meier estimates [20, 23]. Furthermore, we stratified the meta-analysis by model and gender. Based on previous recommendations [23], random-effect models with restricted maximum likelihood estimation for the pooled C statistic, OE ratio, and approximate 95% prediction intervals were used.

We investigated the heterogeneity among the included studies through sensitivity analysis. Several prespecified sensitivity analyses were performed in which we investigated the influence of risk of bias and case-mix difference (e.g. ethnic group, age range and alternative estimated OE methods) on our findings (Supplementary file). All analyses were implemented in R version 3.10 (R Core Team, Vienna, Austria) using the package metamisc [23].

## Results

### Study selection and characteristics

**Fig. 1:**
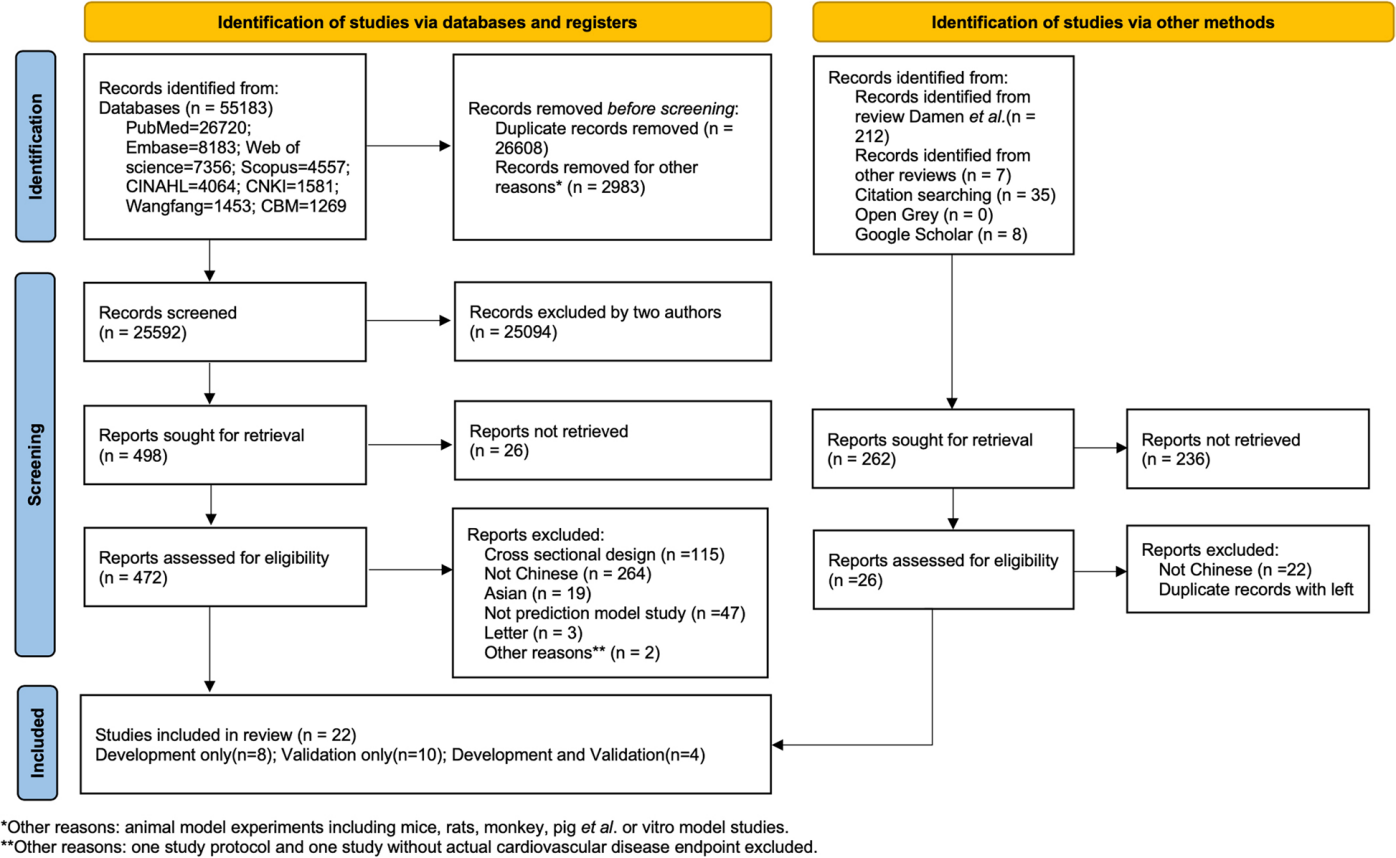
Flow diagram of selected studies

The search strategy identified 55,183 records, of which 26,608 duplicated records and 2983 animal model or in vitro model studies were excluded; then, 25,592 records were screened based on the title and abstract. Two hundred sixty-two records were identified via other methods. In total, 472 full texts were assessed for eligibility, of which 22 studies were included in this review (Fig. 1). Twelve studies [13, 15–18, 27–33] described the development of one or more CVD risk prediction models, and 10 articles [11, 12, 14, 25, 34–39] especially concerned the external validation of one or more risk models. Frequently, four studies [13, 15, 16, 33] described combinations of derived or external validation; therefore, the total number does not sum up to 22. Twenty (90.9%) of the eligible articles were published in English, and 2 reports were written in Chinese.

### Risk of bias

**Fig. 2:**
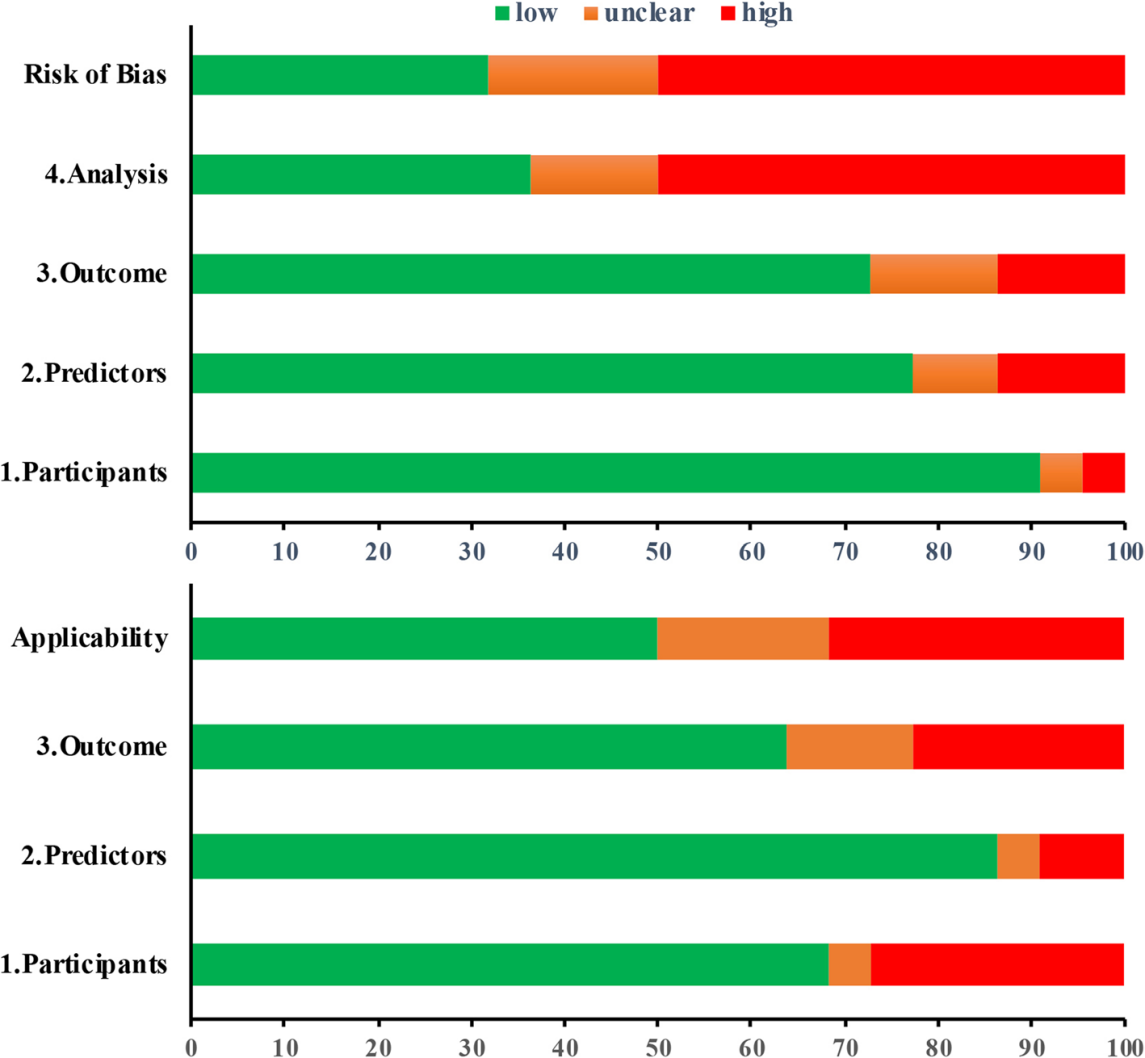
Risk of bias assessment

Most studies were deemed to be of low risk for participant selection and predictors of risk bias. In the outcome domains, the majority of studies reported outcome definitions using ICD codes or WHO criteria, while few studies did not report the outcome definition details. More than half of the validations scored a high risk of bias due to the inadequate handling of missing data. Seven studies scored high concerns of applicability according to inappropriate inclusion criteria, subjectively defined predictors and unclear outcome definitions. A summary of the risk of bias analysis is shown in Fig. 2.

### Development of prediction models

Ten models were formed in 10 distinct papers [13, 15, 17, 18, 27–31, 33], and two articles reported more than two models [16, 32]. Of these, 11 models were developed in the cohort from one region of China (e.g., Beijing, Zhejiang or China Taiwan); there were 7 models derived based on a multicentre cohort including more than two provinces of mainland China (Table 1). In particular, the PREDICT equation [30] was developed based on 400,000 primary care cohorts in New Zealand, including Chinese or other Asian (10.15%) individuals. Fifteen (83.3%) were sex-predictor models. Table 1 shows the year in which the models were published. CVD prediction model development began in 2003 [17], and the latest model was published in 2021 [33]. Thirteen (72.2%) models were published since 2010, while the recruitment year of the cohort had a broad range from 1994 to 2014. The median follow-up time ranged from 3 years to 15.1 years according to the variation in the model prediction horizon. Ten of the selected reports included population-based samples of the general population, whereas 2 studies included people who underwent physical examinations [29] or health management [18], and one study predicted CVD risk for high-risk CVD people [32].

**Table 1:** Characteristics of development studies

Reference	Derivation model	Recruitment years	Median FU time / Prediction horizon	Study Settings	Derivation cohort size	Internal Validation cohort size/method	Age range	Predictors	Outcomes	Modeling Method	Model accessibility	C statistic (95% ***CI***)	Calibration	external validation
Wang 2003 [17] ^a^	10-year Risk model of CVD	1992 ~ 2002	6.1/10y	CMCS	31,728	No	35–64	Age, Gender, TC, SBP, HDL-C, smoking, FG	Fatal or nonfatal CVD	COX regression	Yes/Risk equations	Male 0.78 (0.76–0.81) Female 0.76 (0.72–0.80)	NR	NO
Liu2004 [15]	CHD risk model	1992–1993 1996–1999	10/10y	CMCS	30,121	No	35–64	Age, Gender, TC, BP, HDL-C, smoking, DM	Fatal CHD	COX regression	Yes/Risk equations	Male 0.76 (0.70–0.82) Female 0.74 (0.70–0.78)	Hosmer-Lemeshow test	NO
Zhang2005 [27]	10-year CVD risk prediction score	1974–1980	13.5/10y	Beijing	3000	1400/ random split-sample	18–74	Age, SBP, DBP, TC, BMI, smoking	Fatal or nonfatal CVD	COX regression	Yes/Risk equations	CHD events: training dataset 0.76/validation dataset 0.76; IS events: training dataset 0.72/validation dataset 0.78	Hosmer– Lemeshow test	No
Wu 2006 [16]	10-year Risk prediction model of ICVD	1983–1984	15.1/10y	USA-PRC cohort	9903	No	35–59	Age, Gender, SBP,TC,BMI,smoking, DM	Fatal or nonfatal CVD	COX regression	Yes/Risk Sheet/ online calculator	Optimal model: male 0.80 (0.76–0.83)/female 0.79 (0.76–0.83) simplified model: male 0.79 (0.76–0.83)/female 0.78 (0.75–0.82)	Hosmer– Lemeshow test	Yes
Yang 2016 [13]	China-PAR	1998 2000–2001	12.3/10y	InterASIA MUCA (1998)	21,320	21,320/10*10 cross-validation	35–74	Age, Gender, SBP/Rx, TC, HDL-C, smoking, DM, WC, GR, FHAC, Urbanization	Fatal or nonfatal CVD	COX regression	Yes/Online calculator	Male 0.79 (0.78–0.81) Female 0.81 (0.79–0.82)	Hosmer– Lemeshow test and slope	Yes
Hu 2017 [29]	Cardiovascular death prediction model	1994	8.8/10y	Taiwan	381,963	No	20+	Age, Gender, BMI, smoking, physical activity, anemia, SBP, FG, TC, HDL, LDL, proteinrria, uric acid, CKD, CRP, heart rate, hypertension treatment	CVD death	COX regression	No	0.91 (0.90–0.92)	NR	No
Li 2017 [18] ^a^	Risk prediction model of CVD	2004	3.09/5y	Shandong	50,990	21,853/10*10 cross-validation	20+	Age, Gender, BMI, DM, CKD, abnormal electrocardiogram, smoking, hypertension, dyslipidemia	Fatal or nonfatal CVD	COX regression	NO	Training dataset: male 0.84 (0.82–0.85)/Female 0.90 (0.88–0.91) Validation dataset: male 0.84 (0.81–0.86)/female 0.89 (0.87–0.91)	NR	No
Pylypchuk 2018 [30]	PREDICT equations	2002	4.2/5y	New Zealand	401,752	166,611/geographical split-sample	30–74	Age, Gender, NZDep, smoking history, diabetes, SBP, TC/HDL, OBPLM	Fatal or nonfatal CVD	COX regression	Yes/Risk equations	Male 0.73 (0.72–0.73) Female 0.73 (0.72–0.73)	Calibration slope	No
Li 2020 [31]	Risk prediction model of CVD	2004	10/10y	Taiwan	1481	740/bootstrap resampling	40+	Age, Gender, Marital status, BMI, smoking, physical activity, eGFR, ACR, history of heart disease, history of stroke, ABI	Fatal or nonfatal CVD	COX regression	NO	0.88 (0.83–0.93)	Hosmer– Lemeshow test	No
Yang 2020 [32]	CVD prediction model for high-risk CVD population	2014	3/3y	Zhejiang	19,953	9977/random split-sample	35+	Age, Gender, Family income, smoking, drinking, obesity, WC, TC, TG, LDL, FG, action capability, Self-care ability, Daily activity ability, pain, anxiety, History of hypertension/diabetes/dyslipidemia; Family history of hypertension/ischemic stroke and cerebral infarction; Hypoglycemic drugs use	CVD events	Random forest/CART/ multivariate regression/ NaïveBayes/ Bagged trees /Ada Boost	No	optimal model (random forest) from 6 models: Male 0.82/female 0.68	Hosmer– Lemeshow test	NO
Huang2021 [33]	GBCS prediction model	2003–2008	12/10y	China/Guangzhou	15,000	12,721/10*10 cross-validation	50+	Age, Gender, SBP, antihypertensive medication use, ever smoking, and diabetes status	Fatal or nonfatal CVD	COX regression	Yes/Risk equations	Training dataset: male 0.69 (0.67–0.71)/female 0.73 (0.71–0.74) Validation dataset: male 0.67 (0.65–0.70)/female 0.72 (0.70–0.73)	NR	No
Wang 2015 [28]	CVD lifetime risk model	1992	18/lifetime	CMCS	21,953	No	35–84	SBP/DBP, non-HDL-C, HDL-C, BMI, Diabetes, Smoking	Fatal or nonfatal CVD	Kaplan-Meier method	Yes/Risk sheet	NR	NR	No

*CVD* Cardiovascular disease, *CMCS* Chinese multi-provincial cohort study, *TC* Total cholesterol, *SBP* Systolic blood pressure, *HDL-C* High-density lipoprotein cholesterol, *FG* Fasting blood-glucose, *NR* Not reported, *CHD* Coronary heart disease, *BP* Blood pressure, *DM* Diabetes mellitus, *DBP* Diastolic blood pressure, *BMI* Body mass index, *ICVD* Ischemic cardiovascular disease, *USA-PRC* USA–People’s Republic of China, *China-PAR* Prediction for atherosclerotic cardiovascular disease in China, *InterASIA* International collaborative study of cardiovascular disease in Asia, *MUCA* China Multi-Center Collaborative Study of Cardiovascular Epidemiology, *WC* Waist circumference, *GR* Geographic region, *FHAC* Family history of ASCVD, *LDL* Low-density lipoprotein, *CKD* Chronic kidney disease, *CRP* C-reactive protein, *eGFR* Estimated Glomerular filtration rate, *ACR* Albumin-to-creatinine ratio, *ABI* Ankle– brachial index, *TG* Triglyceride, *CART* Classification and regression tree, *NZDep* New Zealand Index of Socioeconomic Deprivation, *OBPLM* On blood pressure-lowering medications, *GBCS* Guangzhou Biobank cohort study. a: Chinese article

### Predictors in the development papers

The number of predictors was 6–22 [IQR: 6.75–11.25]. Age, smoking, sex, SBP, total cholesterol, diabetes, BMI, and high-density lipoprotein were the most commonly used predictors in the development models. However, lifestyle predictors (e.g., drinking, physical activity, action capability) and ECG factors (e.g., heart rate, abnormal electrocardiogram) were considered in a few models. The incidence of fatal and nonfatal cardiovascular events was defined in nine models, but the definitions of these outcomes showed considerable heterogeneity (Table S3); two models from China Taiwan used CVD death as the follow-up outcome [29, 31]. Fourteen models from eight studies predicted 10-year CVD risk, while less than 5-year CVD risk was predicted in three models, and one model predicted lifetime CVD risk. A Cox proportional hazard regression model was performed to model establishment in 10 articles. One article reported the random forest, classification and regression tree (CART), naïve Bayes, bagged trees, and AdaBoost methods. Nine (50.0%) models provided full risk equations or risk sheets, in which 2 studies also designed the online calculator based on the risk equation. Regarding model performance, the c-statistic or area under the receiver operating characteristic curve was reported in 11 studies. The median c-statistic was similar at 0.77 [range: 0.67–0.84] in men and 0.78 [range: 0.68–0.89] in women. The *Hosmer–Lemeshow* test was the most frequently reported in model calibration, and 5 studies did not report model calibration performance. Internal validation was conducted in 6 studies, most often using a random split of the dataset. Only 2 models were validated in an external cohort.

### External validation of prediction models

A total of 14 studies described 29 validations of 8 models (Supplementary Table 4). Four models were validated more than two times, including Pooled Cohort Equations Goff 2013 (*n* = 11 validations), China-PAR 2016 (*n* = 6), Framingham D’Agostino 2008 (*n* = 5) and Framingham Wilson (*n* = 2) (Table 2). Of the 8 validated models, three models were fully derived in the cohort from China, and 5 models were outside of China. All these models were validated in Chinese and the area, including provinces from mainland China and Hong Kong. Additionally, two studies validated the model in Malaysian Chinese [34, 35] and one in Chinese American [36]. The c-statistic was reported in all the validation studies and ranged from 0.60 to 0.83. While the observed/expected (OE) ratio was reported in a few studies, four studies only reported *Hosmer–Lemeshow* test values.

**Table 2:** Characteristics of validations of included studies

	Framingham	Framingham	PCE	WHO charts for east Asia	Asian equation	China-PAR ^b^	Risk model (Optimal) ^b^	Risk model (Simplied) ^b^
	Wilson1998	D’Agostino2008	Stone2013	WHO2019	Asia2007	Yang2016	Wu2006	Wu2006
	*n* = 2	*n* = 5	*n* = 11	*n* = 2 ^a^	*n* = 1	*n* = 6	*n* = 1	*n* = 1
**Location of the validation cohorts**								
Single-province in mainland China	0	3	2	0	0	4	0	0
Multi-province in mainland China	2	0	6	2	1	2	1	1
China HongKong	0	1	1	0	0	0	0	0
Ethic Chinese	0	1	2	0	0	0	0	0
**Participant age in the validation cohorts**								
Min, Median	30	30	35	40	30	35	35	35
Max, Median	75	74	79	80	75	74	59	59
**Size of the Validation cohorts**								
Sample size, median[range]	27,901 [25,682–30,121]	7157 [438–27,721]	20,886 [425–70,838]	23,329 [27,321–29,337]	25,682	21,631 [3347–70,838]	15,100	15,100
Events,median[range]	366 [191–542]	880 [45–3732]	622 [21–1493]	1070 [1045–1091]	542	1209 [190–3732]	347	347
**Recruitment years of the Validation cohorts**								
< 2000 year	2	2	6	2	1	1	1	1
2001 ~ 2010 year	0	2	4	0	0	5	0	0
> 2010 year	0	1	1	0	0	0	0	0

^a^ one validation for WHO lab and non-lab respectively; ^b^ model derived in Chinese cohort

### Summary of the predictive performance of the externally validated model

We quantitatively synthesized four models validated more than once, and the performance of four models (i.e., WHO chart for East Asia [40], Asian risk model) that was validated only one time is shown in Table S4.

### Calibration

**Fig. 3:**
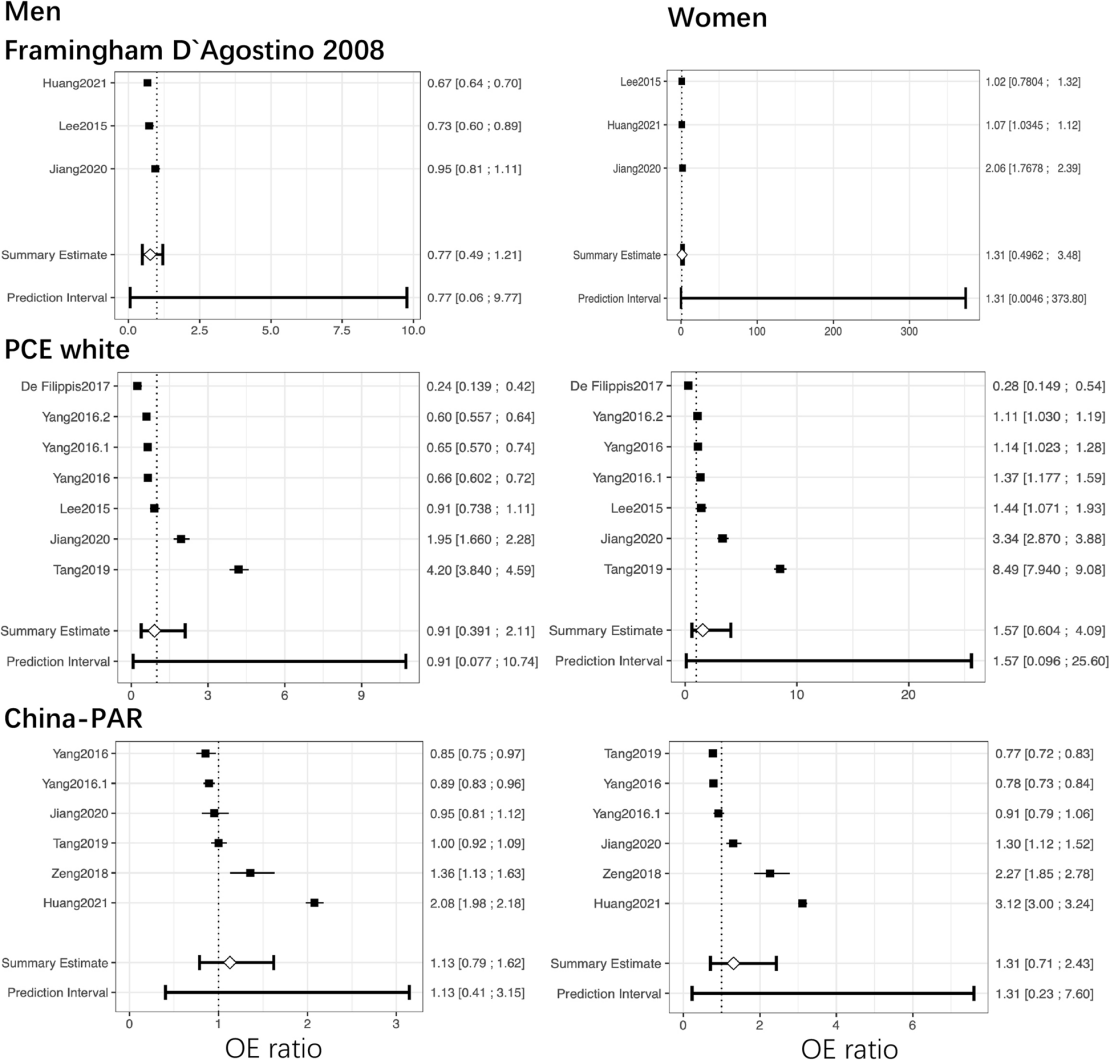
Forest plots of the OE ratio in external validations

Figure 3 shows the summarized estimated calibration of the three models across genders. For the PCE, we excluded 3 validations using the PCE African American model because the AHA guideline [41] advises using the white model for Chinese people, and one validation did not validate separately among genders. Thus, we quantitatively synthetized 7 validations using PCE white. The PCE model showed overprediction in men and underprediction in women with a large range of prediction intervals. For the Framingham D’Agostino model, the number of observed events was lower than the number of predicted events in men and vice versa in women. However, the summarized prediction performance was worst in Framingham Wilson (O/E ratio 0.27 for men and 0.50 for women) compared to other models, which was not shown in the forest plot due to insufficient articles. China-PAR, as a model derived in Chinese, underestimated CVD risk in men and women with a relatively narrow range, similar to the 10-year risk prediction model of ICVD by *Wu* et al. 2006.

### Discrimination

**Fig. 4:**
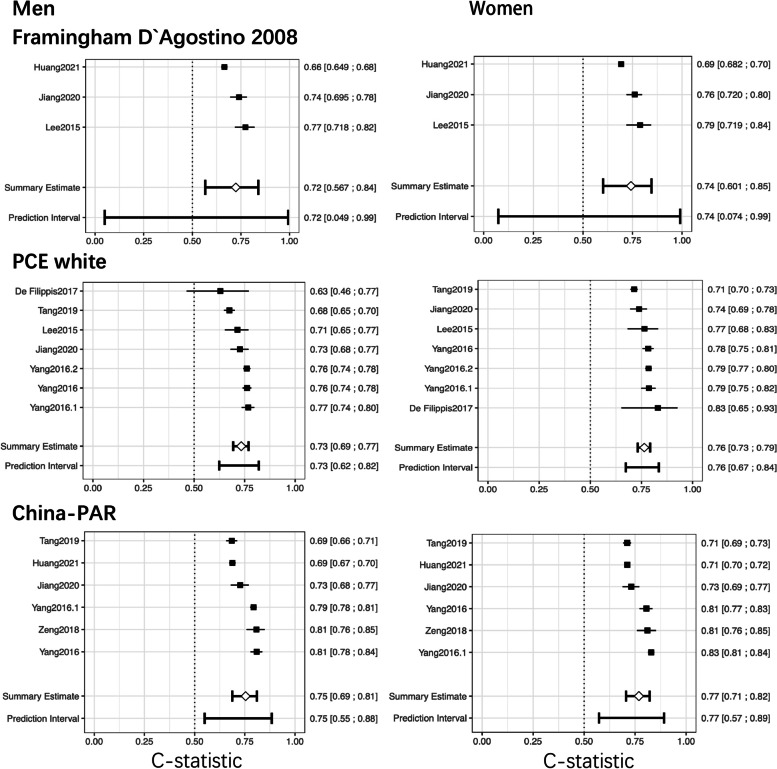
Forest plots of the c-statistic in external validations

The discriminative performance was similar among the eight models. The performance was slightly better for women than for men among the three pooled models (Fig. 4). The pooled prediction performance was better in models derived from Chinese or Asian populations than in those from Western cohorts (Table S4).

### Sensitivity analysis

Sensitivity analyses revealed no effect of study quality on the pooled performance of the models, both for calibration and discrimination. The discrimination for the PCE model decreased after excluding studies with less than 10-year cohorts, while the China-PAR and Framingham D’Agostino model showed no effect. We conducted sensitivity analysis for the PCE model after distinguishing the ethics group of participants in validation studies, and the results showed no effect on calibration but reduced discrimination after excluding the American Chinese cohort. Considering that the model performance may be influenced by the age range of the included participants, we conducted sensitivity analysis after excluding the study by Huang et al. [33] (the lower limit was more than 10 years higher, 50 years vs. 35 years). The results showed improved performance both for calibration and discrimination, as shown in Figs. S1, S2, S3 and S4.

### Reliability and clinical usability of available models

All 23 models derived or validated in the Chinese population were first assessed for reliability according to the criteria mentioned before. Six models (26.1%) met these criteria and were considered reliable (Table S5). Then, these models were assessed for clinical usability through 4 aspects. The Framingham D’Agostino model and PCE had the highest usability score as they met all criteria. China-PAR had high usability with an online calculator with 11 predictors. Other models, such as WHO charts, had higher usability than the Asian equation since the risk chart was more convenient than the equation.

## Discussion

### Summary of evidence

Although many cardiovascular disease prediction models have been developed at home and abroad [9, 10], none of them systematically review the applicability and performance of the model among the Chinese, which causes some confusion in clinical practice. Our work complements this evidence from model development and external validation aspects. Finally, twenty-two studies were eligible, of which 18 models were developed, and 3 models also conducted external validation; 5 models were derived from foreign countries validated in Chinese. The Framingham and the PCEs are the most studied tools. China-PAR has been externally validated most frequently as one of the models derived from Chinese. Additionally, the WHO lab or nonlab-based charts for East Asia and Asia cardiovascular risk prediction tools were validated only once. Discrimination was acceptable and similar for men and women among these models. The calibration estimates changed substantially from one population to another. The China-PAR showed good calibration both in men and women, while the Framingham Wilson model showed serious overestimation. Five external models were validated less than two times, so we could not compare them and draw absolute conclusions.

### Model development and validation

A number of studies developed new risk prediction models for cardiovascular disease in China, although 72.2% of the models were published after 2015. As we know, most models were developed in the European and Northern American populations [9], and it is urgent and necessary to construct a risk prediction model based on the Chinese population to guide CVD prevention in China. However, we found much variability in age range, predictors, and predicted outcomes among development studies. With respect to the outcome definitions, we found similar manifestations with whole risk prediction models worldwide [9]. The heterogeneity of definition for fatal or nonfatal CVD between studies was significant, while different definitions may lead to disparate estimated prediction effects and consequently indicate various treatment strategies based on these models [6]. Thus, this discourages head-to-head comparisons or quantitative integrations between studies, as well as clinical applications. New studies reporting on prediction models should adhere to the transparent reporting of a multivariable prediction model for individual prognosis or diagnosis (TRIPOD) guidelines [42, 43] to guarantee uniformity.

We also found an exciting phenomenon that an increasing number of risk prediction models derived from international populations contain Chinese, for instance, WHO risk charts based on 85 international cohorts [40] and PREDICT equations developed for 182 countries [30]. This gave more options for CVD risk prediction in China. However, the number of validation studies in the Chinese population is not sufficient, and we could not draw firm recommendation conclusions for any of them. We advise that more validation studies exist of these models to enrich the evidence.

In addition, internal and external validation are both important for modelling studies. Approximately half of the models conducted internal validation, whereas rare external validations were conducted. This suggested that we made a great effort to develop new models rather than validate, tailor, and improve existing CVD risk prediction models. In contrast, external validation is needed to ensure the transportability of a prediction model. Although two Chinese models were externally validated in another Chinese cohort, CVD risk varies geographically, and the major contributing risk factors were different across regions in China (i.e., a higher hypertension ratio in northern China, lowest ratio of metabolic and physical activity in northwestern China and lowest ratio of smoking and alcohol in northeastern China) [44]. This means that one model derived from a single region is imprecise for evaluating the real risk in another area. Despite China-PAR being derived from two multiprovince cohorts, it still overestimated the real risk of the Inner Mongolia population or the elderly in the validation studies [33, 39]. However, pooled C statistics and the estimated OE ratio indicated the good performance of the China-PAR in China. This result indicates that the China-PAR may be a better choice for CVD risk prediction in China. Compared with the performance of Framingham prediction models in previous studies [45], we found similar results that both Framingham Wilson and Framingham D’Agostino overestimate cardiovascular disease risk. Furthermore, we also found that the Framingham D’Agostino overestimated the cardiovascular disease risk in men and underestimated it in women. This tendency was also found in PCE. Thus, the applicability of these two models in clinical practice was limited by their calibration.

In addition, the sensitivity analysis showed reduced discrimination after excluding the American Chinese cohort for the PCE model, and PCE derived from the multi-ethnic population could be a potential reason. We extrapolated the OE ratio to 10 years using the equation based on Poisson distribution [20] for studies with a prediction horizon less than 10 years, and sensitivity analysis was performed after excluding these extrapolated studies to clarify this estimation on the pooled performance of these models. The results indicated that the characteristics of the validation cohort should be comparable with the derived cohort to improve the accuracy of prediction.

### Implications for clinical practice

A number of guidelines have recommended that cardiovascular prevention and treatment be based on risk assessment [41, 46]. The clinical usability of the risk prediction model requires not only acceptable external validation performance but is also easy to use, such as the accessibility of predictors and the availability of a full regression equation or online calculators [24]. However, there are no well-established reliability and clinical usability evaluation tools for prediction models. We designed three criteria for reliability and four criteria for clinical usability according to expert consultation based on previous studies by *Baart SJ* et al. [24] and *Barzi F* et al. [25]. Of all 23 models, only 6 (26.1%) models met the reliability criteria. When further evaluated for clinical usability, only the Framingham D’Agostino and PCE models met all criteria, rendering them more appealing for clinical practice. The China-PAR model derived from the Chinese population was recommended by several Chinese CVD prevention guidelines and met all criteria apart from fewer than 10 predictors (11 predictors). Future research should focus on externally validating these three models or tailoring these models to local settings to gain better prediction performance.

### Limitations

This study has several limitations. First, we had to rely on the reports of authors for primary validation studies, and therefore we had to exclude relevant validations from the final meta-analysis because we could not obtain unreported information from the authors. We excluded two validations in the pooled performance analysis of the Framingham model and one validation of the PCE model, which makes it difficult to argue whether the predictive performance of models will change or not when all validations are included. Second, the number of observed and expected events and the total participant numbers were needed to estimate the pooled OE ratio. Two validations only reported bar charts of predicted or actual events by 10 deciles of predicted risk. We had to infer the OE ratio of each decile through the prediction chart without proofreading the authors. Furthermore, we performed a sensitivity analysis for China-PAR to verify the stability of the results when excluding those inferred validations. The same analysis was not conducted in the Framingham D’Agostino model because of the lower number of validations. Third, we did not perform meta-regression analysis to explore potential heterogeneity factors for each model due to the limitation of the number of validation studies. Generally, at least 10 reports were needed for meta-regression to obtain valid results [20]. Thus, we investigated the heterogeneity through prespecified sensitivity analyses investigating the influence of risk of bias and case-mix difference on our findings. Fourth, we initially evaluated reliability and clinical usability for available models based on criteria designed based on previous publications and expert consultation owing to the lack of well-established tools reported. This may affect the assessment accuracy and requires further study. Fifth, we did not search the publications in languages apart from English and Chinese because of limited resources, which may lead to potential bias for the study findings.

## Conclusions

In conclusion, there is an increasing trend for cardiovascular disease prediction models developed and validated in the Chinese population, but the validation study was still insufficient. Only the pooled estimated calibration and discrimination of Framingham D’Agostino, PCE and China-PAR were calculated due to the insufficient number of validated model studies, of which China-PAR showed better performance than the other two models. Thus, the usefulness of most CVD prediction models remains unclear due to incomplete external validation and head-to-head comparison based on current search strategies. Future research should focus on externally validating or tailoring these models to local settings.

## Supplementary Information

**Additional file 1: Supplementary Table 1.** Search strategy for Pubmed. **Supplementary Table 2.** Items for data extraction of development and validation studies. **Supplementary Table 3.** Outcome definition of development studies. **Supplementary Table 4.** Characteristics of validations. **Supplementary Table 5.** Clinical usability of models that met the reliability criteria. **Fig. S1.** Sensitivity analyses considering risk of bias (A and B). China-PAR validations excluded studies by Jiang 2020 and Zeng 2008; PCE white validations excluded studies by Jiang 2020 and Lee 2015. PCE: Pooled Cohorts Equations, OE: observed expected. **Fig. S2.** Sensitivity analyses considering less than 10-year prediction horizons (A and B). China-PAR and PEC validations both excluded studies by Yang 2016(CIMIC cohort), Jiang 2020 and Tang 2019; Framingham D’Agostino model validations excluded studies by Jiang 2020. PCE: Pooled Cohorts Equations, OE: observed expected. **Fig. S3.** Sensitivity analyses considering age group for China-PAR model (A and B). OE: observed expected. **Fig. S4.** Sensitivity analyses considering ethic group for PCE model. PCE: Pooled Cohorts Equations, OE: observed expected.

## Data Availability

All data analysed during this study are included in this article and its supplementary information files.

## Abbreviations

CVD: Cardiovascular disease
CNKI: China National Knowledge Infrastructure
PROBAST: Prediction Model Risk of Bias Assessment Tool
PCE: Pooled cohort equations
China-PAR: Prediction for atherosclerotic cardiovascular disease Risk in China
PROSPERO: International prospective register of systematic reviews
PRISMA: Preferred reporting items for systematic reviews and meta-analyses
CHARMS checklist: Checklist for appraisal and data extraction for systematic reviews of prediction modelling studies
OE: Observed expected
SBP: Systolic blood pressure
BMI: Body mass index
ECG: Electrocardiogram
CART: Classification and regression tree
TRIPOD: Transparent reporting of a multivariable prediction model for individual prognosis or diagnosis
