# Supplementary material for: Cardiovascular disease risk prediction models in the Chinese population- a systematic review and meta-analysis
Source: BMC Public Health. 2022 Aug 24;22:1608. doi: 10.1186/s12889-022-13995-z (PMC9400257; doi:10.1186/s12889-022-13995-z)
Supplement: Supplementary file 1 — Additional file 1: Supplementary Table 1. Search strategy for Pubmed. Supplementary Table 2. Items for data extraction of development and validation studies. Supplementary Table 3. Outcome definition of development studies. Supplementary Table 4. Characteristics of validations. Supplementary Table 5. Clinical usability of models that met the reliability criteria. Fig. S1. Sensitivity analyses considering risk of bias (A and B). China-PAR validations excluded studies by Jiang 2020 and Zeng 2008; PCE white validations excluded studies by Jiang 2020 and Lee 2015. PCE: Pooled Cohorts Equations, OE: observed expected. Fig. S2. Sensitivity analyses considering less than 10-year prediction horizons (A and B). China-PAR and PEC validations both excluded studies by Yang 2016(CIMIC cohort), Jiang 2020 and Tang 2019; Framingham D’Agostino model validations excluded studies by Jiang 2020. PCE: Pooled Cohorts Equations, OE: observed expected. Fig. S3. Sensitivity analyses considering age group for China-PAR model (A and B). OE: observed expected. Fig. S4. Sensitivity analyses considering ethic group for PCE model. PCE: Pooled Cohorts Equations, OE: observed expected. [file 12889_2022_13995_MOESM1_ESM.docx]

Supplementary Table 1. Search strategy for Pubmed

| **Concept Step Search Strategy** | | | | |
| --- | --- | --- | --- | --- |
| Population = General population based on primary care ^1^ | | | | |
|  | 1 | | | "Primary health care"[Mesh] |
|  | 2 | | | ("public health"[Title/Abstract]) OR (community [Title/Abstract])) OR (care, primary health[Title/Abstract]) OR (health care, primary[Title/Abstract]) OR (primary healthcare[Title/Abstract]) OR (healthcare, primary[Title/Abstract]) OR (primary care[Title/Abstract]) OR (care, primary) |
|  | 3 | | | 1 OR 2 |
| Outcome = cardiovascular diseases ^2^ | | | | |
|  | 4 | | "Cardiovascular Diseases "[Mesh] | |
|  | 5 | | (cardiovascular disease*[Title/Abstract]) OR (disease, cardiovascular[Title/Abstract]) OR (CVD[Title/Abstract]) OR (cardiovascular*[Title/Abstract]) OR (CV[Title/Abstract]) OR (heart disease*[Title/Abstract]) OR (cardiac disease*[Title/Abstract]) OR (cardiac disorder*[Title/Abstract]) OR (coronary disease[Title/Abstract]) OR (coronary heart disease*[Title/Abstract]) OR (CHD[Title/Abstract]) OR (cerebrovascular*[Title/Abstract]) OR (stroke[Title/Abstract]) | |
|  | 6 | | 4 OR 5 | |
| Study type = prediction modelling studies ^3^ | | | | |
|  | 7 | (predict*[Title/Abstract]) OR (progn*[Title/Abstract]) OR (algorithm*[Title/Abstract]) OR (risk predic*[Title/Abstract]) OR (risk scor*[Title/Abstract]) OR (risk table[Title/Abstract]) OR (risk chart*[Title/Abstract]) OR (risk equation*[Title/Abstract]) OR (risk factor*[Title/Abstract]) OR (risk calculation[Title/Abstract]) OR (risk assessment[Title/Abstract]) | | |
|  | 8 | 3 AND 6 AND 7 | | |
| Human filter ^4^ | 9 | Animals [MH] NOT Humans [MH] | | |
|  | 10 | 8 NOT 9 | | |
| Language filter | 11 | English OR Chinese | | |
|  | 12 | 10 AND 11 | | |
| Age filter | 13 | 12 AND all adult (19+years) | | |

References:

1. Damen J A , Pajouheshnia R , Heus P , et al. Performance of the Framingham risk models and pooled cohort equations for predicting 10-year risk of cardiovascular disease: a systematic review and meta-analysis[J]. BMC Medicine, 2019, 17.

2. Damen J , Hooft L , Schuit E , et al. Prediction models for cardiovascular disease risk in the general population: systematic review[J]. BMJ, 2016, 353:i2416.

3. Bellou V, Belbasis L, Konstantinidis AK, et al. Prognostic models for outcome prediction in patients with chronic obstructive pulmonary disease: systematic review and critical appraisal. BMJ (Clinical research ed) 2019;367:l5358. doi: 10.1136/bmj.l5358 [published Online First: 2019/10/06]

4. Cochrane handbook for systematic reviews of interventions: The Cochrane Collaboration; 2011. Available from: <http://handbook-5-1cochraneorg/>

Supplementary Table 2 Items for data extraction of development and validation studies

| **Category** | **Item** | **Description / examples** |
| --- | --- | --- |
| Development and validation studies | Model name | Framingham Wilson, PCE; China-PAR; men or women. |
|  | Study type | Only development of a new model; only external validation; both develop and external validation. |
|  | Study design | Cohort, randomized controlled trial |
|  | Eligibility criteria for participants | Age, (exclusion of) comorbidities, treatment, race. |
|  | Study dates | Inclusion dates, end of follow-up, follow-up time. |
|  | Prediction horizon | Time period for which predictions were made, e.g. 10 years. |
|  | Location | Country and continent. |
|  | Predicted outcome | Full definition, including ICD-codes. |
| Validation studies | Sample size | Number of participants, number of events, Kaplan-Meier l0-year survival probability. |
|  | Performance | C-statistic, 10-year total observed/expected ratio, standard error, 95% confidence intervals, calibration plot, calibration table. Performance of the original model was extracted. |
| Development studies | Modelling method | Derivation cohort size; Cox regression, Random forest or other methods. |
|  | Predictors | Full definition, measurement method, blinding of measurements. |
|  | Model presentation | Risk equations, risk charts or tables. |
|  | Internal validation | Internal validation cohort size and method. |
|  | External validation | External validation cohort information |
|  | Performance | C-statistic, 95% confidence intervals, calibration plot, calibration table, Hosmer– Lemeshow test value. |

Supplementary Table 3 Outcome definition of development studies

| **Reference** | **Derivation model** | **Outcome category** | **outcome definition** |
| --- | --- | --- | --- |
| Wang 2003 | 10-year Risk model of CVD | Fatal or nonfatal CVD | Acute CHD events (myocardial infarction / Sudden death from coronary heart disease/Other coronary heart disease deaths, acute ischemic stroke events |
| Liu2004 | CHD risk model | Fatal CHD | Myocardial infarction / Sudden death from coronary heart disease (acute myocardial infarction, sudden death, and other coronary deaths) |
| Zhang2005 | 10-year CVD risk prediction score | Fatal or nonfatal CVD | Fatal or nonfatal CHD (angina pectoris and myocardial infarction (ICD- 9 Codes: 410–414.9; sudden death); Fatal or nonfatal stoke hemorrhagic and ischemic stroke (ICD-9 Codes: 430–438), stroke death. |
| Wu 2006 | 10-year Risk prediction model of ICVD | Fatal or nonfatal CVD | Myocardial infarction / Sudden death from coronary heart disease/Other coronary heart disease deaths/ Ischemic stroke (ICD-9 433 and434) (TIA and other causes of cerebrovascular disease are not included). |
| Yang 2016 | China-PAR | Fatal or nonfatal CVD | Nonfatal acute MI or CHD death or fatal or nonfatal stroke. Acute MI was identified as a change in biochemical markers of myocardial necrosis accompanied by ischemic symptoms, pathological Q waves, ST-segment elevation or depression, or coronary intervention.18 CHD death included all fatal events resulting from MI or other coronary deaths. Stroke included clinical signs and symptoms of sub- arachnoid or intracerebral hemorrhage or cerebral infarction, which were rapidly developing signs of focal (or global) disturbances in cerebral function lasting >24 hours without an apparent nonvascular cause. |
| Hu 2017 | Cardiovascular death prediction model | CVD death | CVD death was matched to the national death file, but without details. |
| Li 2017 | Risk prediction model of CVD | Fatal or nonfatal CVD | Fatal or nonfatal CVD contains ICD-10(I11/I120-127/I152) and ICD-10(I60-I69). |
| Pylypchuk 2018 | PREDICT equations | Fatal or nonfatal CVD | Ischemic heart disease (including angina); ischemic or hemorrhagic cerebrovascular events (including transient ischemic attacks); or peripheral vascular disease, congestive heart failure, or other ischemic cardiovascular disease deaths. |
| Li 2020 | Risk prediction model of CVD | CVD death | CVD death contains ICD-9 390-459, ICD-10 I00-I99+diabetes ICD9 250, ICD10 E10-14+kidney disease ICD9-580-589, ICD10-N00-29) |
| Yang 2020 | CVD prediction model for high-risk CVD population | Fatal or nonfatal CVD | Events related to atherosclerosis (angina pectoris, stroke, acute myocardial infarction, coronary heart disease, heart failure, arrhythmia and else). |
| Huang2021 | GBCS prediction model | Fatal or nonfatal CVD | CVD included CHD (ICD-10, I20–I25), cerebrovascular events (ICD-10, I60–I69), peripheral artery disease (ICD-10, I73), and heart failure (ICD-10, I50). |
| Wang 2015 | CVD lifetime risk | Fatal or nonfatal CVD | Acute coronary events included acute myocardial infarction, sudden coronary death, and other coronary deaths (ICD-9 codes 410 and 414 or ICD-10 codes I21 and I25) and acute stroke events including subarachnoid hemorrhage, intracerebral hemorrhage, or cerebral infarction (ICD-9 codes 430-434 and 436 or ICD-10 codes I60-I64). |

Notes: CVD, cardiovascular disease; CHD, coronary heart disease; ICD, International Classification of Diseases; TIA, transient ischemic attack; China-PAR, prediction for atherosclerotic cardiovascular disease in China; GBCS, Guangzhou Biobank cohort study.

Reference were same to the maintext.

Supplementary Table 4 Characteristics of validations

| **Reference** | **Validated model** | **Recruitment years** | **Median FU/Prediction horizon** | **Cohort** | **Country** | **Predicted outcome** | **N events / n participants (%)** | **Mean age (range)** | **C (SE)** | **OE (SE)** |
| --- | --- | --- | --- | --- | --- | --- | --- | --- | --- | --- |
| Wu2006 | 10-year Risk prediction model of ICVD-male | 1993-1994 | 10/10 | MUCA Cohort II | China/mainland China((Beijing/GuangZhou) | Fatal or nonfatal CVD | 206/6998(2.9) | 46.4(35-59) | 0.796(0.016) | 0.962(0.067) |
|  | 10-year Risk prediction model of ICVD -female |  |  |  |  |  | 141/8102(1.7) | 46.5(35-59) | 0.791(0.019) | 1.143(0.095) |
|  | Simplified point score-male |  |  |  |  |  | 206/6998(2.9) | 46.4(35-59) | 0.792(0.016) | 0.972(0.067) |
|  | Simplified point score-female |  |  |  |  |  | 141/8102(1.7) | 46.5(35-59) | 0.783(0.019) | 1.144(0.096) |
| Liu 2004 | Framingham-Wilson-male | 1992-1993/1996-1999 | 10/10 | CMCS | China/mainland China | Fatal or nonfatal CHD | 137/16065(1.5) | 47.4(35-64) | 0.705(0.022) | 0.296(0.025)^*^ |
|  | Framingham-Wilson-famale |  |  |  |  |  | 54/14056(0.6) | 46.3(35-64) | 0.742(0.034) | 0.481(0.067)^*^ |
| Chia2015 | Framingham-2008 | 1998 | 10/10 | Patients from an urban primary care clinic | Malaysia /Chinese | Fatal or nonfatal CVD | 45/438(10.3) | 56.9(30+) | 0.60(0.044) | 0.441(0.062) |
| Chia 2014 | PCE white | 1998-1998 | 10/10 | Patients from outpatient primary care clinic of University Malaya Medical Centre | Malaysia /Chinese | Fatal or nonfatal CVD | 21/425(4.9) | 57.5(40-79) | 0.625(0.062) | 0.457(0.097) |
| Huang2021 | China-PAR male | 2003-2008 | 12/10 | GBCS | China/Guangzhou | Fatal or nonfatal CVD | 1369/7703(17.8) | 64.14(50+) | 0.668(0.008) | 2.079(0.051) |
|  | China-PAR female |  |  |  |  |  | 2363/20018(11.8) | 60.89(50+) | 0.711(0.006) | 3.526(0.07) |
|  | Framingham-2008-male |  |  |  |  |  | 1369/7703(17.8) | 64.14(50+) | 0.665(0.008) | 0.669(0.016) |
|  | Framingham-2008-female |  |  |  |  |  | 2363/20018(11.8) | 60.89(50+) | 0.692(0.005) | 1.074(0.021) |
| Jiang 2020 | China-PAR male | 2010-2012 | 7.05/5 | Participants from Kashi and Yili | China/Xinjiang | Fatal or nonfatal CVD | 137/1508(9.1) | 53.83(40-74) | 0.727(0.023) | 0.953(0.078)^*^ |
|  | China-PAR female |  |  |  |  |  | 149/1839(8.4) | 52.10(40-74) | 0.731(0.021) | 1.295(0.102)^*^ |
|  | Framingham-2008-male |  |  |  |  |  | 137/1508(9.1) | 53.83(40-74) | 0.740(0.022) | 0.939(0.078)^*^ |
|  | Framingham-2008-female |  |  |  |  |  | 149/1839(8.4) | 52.10(40-74) | 0.761(0.020) | 2.056(0.162)^*^ |
|  | PCE male white |  |  |  |  |  | 137/1508(9.1) | 53.83(40-74) | 0.727(0.023) | 1.902(0.159)^*^ |
|  | PCE female white |  |  |  |  |  | 149/1839(8.4) | 52.10(40-74) | 0.738(0.021) | 3.333(0.262)^*^ |
| Lee 2015 | PCE male white | 1995-2004 | 10/10 | Hong Kong Cardiovascular Risk Factor Prevalence Study (CRISPS) cohort | China/HongKong | Fatal or nonfatal CVD | 80/679 (11.8) | 55.8 (40-74) | 0.714(0.030) | 1.054 (0.102) |
|  | PCE female white |  |  |  |  |  | 42/797 (5.3) | 53.4 (40-74) | 0.765(0.038) | 1.438 (0.191) |
|  | Framingham-2008-male |  |  |  |  |  | 86/771(11.2) | NR (30-74) | 0.773(0.026) | 0.732(0.074) |
|  | Framingham-2008-female |  |  |  |  |  | 52/917(5.67) | NR (30-74) | 0.788(0.032) | 1.016(0.137) |
| Li 2021 | WHO-Lab male | 1992-2001 | 10/10 | China MUCA(1992-1994) China MUCA(1998)  InterASIA | China/mianland | Fatal or nonfatal CVD | 610/12958(4.7) | 54.29/NR | 0.759(0.010) | 0.629(0.025) |
|  | WHO-Lab female |  |  |  |  |  | 435/14363(3.0) | 53.74/NR | 0.752(0.012) | 0.579(0.027) |
|  | WHO-non Lab male |  |  |  |  |  | 642/13962(4.6) | 54.29/NR | 0.762(0.010) | 0.608(0.024) |
|  | WHO-non Lab female |  |  |  |  |  | 449/15375(2.9) | 53.74/NR | 0.754(0.012) | 0.539(0.025) |
| Peng 2014 | Framingham-2008 | 2002 | 10/10 | Inner Mongolia | China/ Inner Mongolia | Fatal or nonfatal CVD | 200/2589(7.7) | NR | 0.810(0.016) | 0.941(0.064) |
| Tang2019 | PCE-male white | 2008-2010 | 6.44/5 | The FangShan Cohort Study(FCS) | China/Beijing | Fatal or nonfatal CVD | 418/3578(11.7) | 55.5(40-79) | 0.675(0.014) | 4.202(0.193)^*^ |
|  | PCE female white |  |  |  |  |  | 749/7591(9.9) | 55.1(40-79) | 0.714(0.009) | 8.475(0.295) |
|  | China-PAR male |  |  |  |  |  | 418/3578(11.7) | 55.5(40-79) | 0.685 (0.014) | 1.020(0.046) |
|  | China-PAR female |  |  |  |  |  | 749/7591(9.9) | 55.1(40-79) | 0.711(0.010) | 0.773(0.027) |
| Zeng2018 | China-PAR male | 2002 | 9.2/10 | Participants from 32 villages | China/ nner Mongolian | Fatal or nonfatal CVD | 103/1056(9.8) | 46.4(NR) | 0.808(0.023) | 1.424(0.133) |
|  | China-PAR female |  |  |  |  |  | 87/1533(5.7) | 46.4(NR) | 0.810(0.024) | 1.831(0.213) |
| Yang 2016 | China-PAR male | 1992-1994 | 17.1/10 | China MUCA (1992) | China | Fatal or nonfatal CVD | 216/6565 (3.3) | 46.5(35-59) | 0.809(0.015) | 0.855(0.057) |
|  | China-PAR male | 2007-2008 | 5.9/5 | CIMIC |  | Fatal or nonfatal CVD | 755/26872 (2.8) | 55.3(35-74) | 0.793(0.008) | 0.893(0.032) |
|  | China-PAR female | 1992-1994 | 17.1/10 | China MUCA (1992) |  | Fatal or nonfatal CVD | 168/7558 (2.2) | 46.6 (35-59) | 0.829(0.016) | 0.914(0.070) |
|  | China-PAR female | 2007-2008 | 5.9/5 | CIMIC |  | Fatal or nonfatal CVD | 738/43966 (1.7) | 53.9 (35-74) | 0.805(0.008) | 0.783(0.029) |
|  | PCE male white | 1998-2001 | 12.3/10 | InterASIA and MUCA (1998) |  | Fatal or nonfatal CVD | 451/10334 (4.4) | 48.8(35-74) | 0.762 (0.011) | 0.657 (0.029) |
|  | PCE male white | 1992-1994 | 17.1/10 | China MUCA (1992) |  | Fatal or nonfatal CVD | 216/6565 (3.3) | 46.5(35-59) | 0.768 (0.016) | 0.649 (0.043) |
|  | PCE male white | 2007-2008 | 5.9/5 | CIMIC |  | Fatal or nonfatal CVD | 755/26872 (2.8) | 55.3(35-74) | 0.761 (0.009) | 0.603(0.023)^*^ |
|  | PCE female white | 1998-2001 | 12.3/10 | InterASIA and MUCA (1998) |  | Fatal or nonfatal CVD | 285/10986 (2.6) | 48.4(35-74) | 0.783 (0.014) | 1.145 (0.062) |
|  | PCE female white | 1992-1994 | 17.1/10 | China MUCA (1992) |  | Fatal or nonfatal CVD | 168/7558 (2.2) | 46.6(35-59) | 0.786 (0.018) | 1.368 (0.104) |
|  | PCE female white | 2007-2008 | 5.9/5 | CIMIC |  | Fatal or nonfatal CVD | 738/43966 (1.7) | 53.9 (35-74) | 0.785 (0.008) | 1.110 (0.041)^*^ |
|  | PCE male African American | 1998-2001 | 12.3/10 | InterASIA and MUCA (1998) |  | Fatal or nonfatal CVD | 451/10334 (4.4) | 48.8 (35-74) | 0.769 (0.011) | 0.562 (0.025) |
|  | PCE male African American | 1992-1994 | 17.1/10 | China MUCA (1992) |  | Fatal or nonfatal CVD | 216/6565 (3.3) | 46.5 (35-59) | 0.790 (0.016) | 0.482 (0.032) |
|  | PCE male African American | 2007-2008 | 5.9/5 | CIMIC |  | Fatal or nonfatal CVD | 755/26872 (2.8) | 55.3 (35-74) | 0.750 (0.008) | 0.571 (0.021)^*^ |
|  | PCE female African American | 1998-2001 | 12.3/10 | InterASIA and MUCA (1998) |  | Fatal or nonfatal CVD | 285/10986 (2.6) | 48.4 (35-74) | 0.796 (0.013) | 0.715 (0.040) |
|  | PCE female African American | 1992-1994 | 17.1/10 | China MUCA (1992) |  | Fatal or nonfatal CVD | 168/7558 (2.2) | 46.6 (35-59) | 0.807 (0.017) | 0.794 (0.061) |
|  | PCE female African American | 2007-2008 | 5.9/5 | CIMIC |  | Fatal or nonfatal CVD | 738/43966 (1.7) | 53.9 (35-74) | 0.792(0.008) | 0.699 (0.026)^*^ |
| De Filippis 2017 | PCE male | 2000-2002 | NR/10 | MESA study | United States/Chinese | Fatal or nonfatal CVD | 12/371(3.2) | NR (45-79) | 0.63(0.018) | 0.241(0.069) |
|  | PCE female |  |  |  |  | Fatal or nonfatal CVD | 9/392(2.3) | NR (45-79) | 0.83 (0.069) | 0.284(0.094) |
| Asia2007 | Framingham-Wilson-male | 1974-1993 | 8.3/8 | APCSC/China cohort | China | Fatal or nonfatal CVD | 418/15046(2.8) | 47(30-75) | 0.75(0.012) | 0.266(0.013)^*^ |
|  | Framingham-Wilson-female |  |  |  |  | Fatal or nonfatal CVD | 124/10636(1.2) | 46(30-75) | 0.79(0.020) | 0.495(0.044)^*^ |
|  | Asian equation-male |  |  |  |  | Fatal or nonfatal CVD | 418/15046(2.8) | 47(30-75) | 0.76(0.012) | 0.901(0.043)^*^ |
|  | Asian equation-female |  |  |  |  | Fatal or nonfatal CVD | 124/10636(1.2) | 46(30-75) | 0.80(0.020) | 0.909(0.081)^*^ |

Notes: FU, follow-up; N, number; C, c-statistic; OE: observed/expected ratio; SE: standard error; ICVD, ischemic cardiovascular disease; MUCA, China Multi-Center Collaborative Study of Cardiovascular Epidemiology; CVD, cardiovascular disease; CMCS, Chinese multi-provincial cohort study; CHD, coronary heart disease; PCE, pooled Cohort Equations; China-PAR, prediction for atherosclerotic cardiovascular disease in China; GBCS, Guangzhou Biobank cohort study; InterASIA, international collaborative study of cardiovascular disease in Asia; CIMIC, community intervention of metabolic syndrome in China & Chinese Family Health Study; MESA, Multi-Ethnic Study of Atherosclerosis; APCSC, Asia Pacific Cohort Studies Collaboration; NR: Not reported.

* OE ratio and corresponding SE extrapolated to 10 years; Reference were same to the miantext.

Supplementary Table 5. Clinical usability of models that met the reliability criteria

| **Model name** | **Author-year** | **≤10 predictors** | **≤ 1medical resource** | **Full equation/ Risk chart** | **Online calculator** |
| --- | --- | --- | --- | --- | --- |
| Framingham | D`Agostino 2008 | ✓ | ✓ | ✓/✓ | ✓ |
| Pooled Cohort Equations | Goff 2013 | ✓ | ✓ | ✓/✕ | ✓ |
| WHO charts for east Asia (Lab) | WHO2019 | ✓ | ✓ | ✕/✓ | ✕ |
| WHO charts for east Asia (non-Lab) | WHO2019 | ✓ | ✓ | ✕/✓ | ✕ |
| Asian equation | Asia 2007 | ✓ | ✓ | ✓/✕ | ✕ |
| China-PAR | Yang 2016 | ✕ | ✓ | ✓/✕ | ✓ |

Notes: China-PAR, prediction for atherosclerotic cardiovascular disease in China; reference were same in the article

**Sensitivity analysis**

We performed several sensitivity analyses to clarify the heterogeneity. No summary statistics were reported for 10-year Risk prediction model of ICVD and Simplified point score by Wu 2006, Framingham-Wilson model, WHO risk chart, Asian equation because of the low number of validations. Thus, we just performed analysis for external validations of China-PAR, PCE and Framingham D`Agostino model.

Firstly, we excluded all external validations with high risk of bias for at least one domain to clarify the effect of study quality on pooled performance of the models. The results showed no effect on both calibration and discrimination. Two studies with high risk of bias for Framingham D`Agostino model validation, and one research left after excluded these two studies, thus no plot derived for Framingham D`Agostino model (Figure S1).

Secondly, since these three models all with prediction horizon of 10 year, but several validation cohorts with less than 10-year follow-up. Thus, we extrapolated observed event risk (PO) and expected event risk (PE) separately to 10 years using the equation based on Poisson distribution. So, further analysis performed after excluding studies with validation cohort less than 10 year to clarify this estimation on the pooled performance of these models. The results showed the discrimination for PCE model decreased after excluding studies with extrapolating to 10-year OE ratio, while China-PAR and Framingham D`Agostino model showed no effect. (Figure S2)

Thirdly, considering that the model performance may be influenced by age range or ethic group of included participants, we conducted sensitivity analysis. The age range of participants in the validation cohort of PCE and Framingham D`Agostino model was comparable, and the age of validation cohort participants of China-PAR was similar apart from Huang et al. (15 years older than the lower limit). So, we performed sensitivity analysis after excluding Huang et al. study for China-PAR model (Figure S3). Study by De Filippis 2017 included Chinese American participants, and other validations were all Chinese, so we performed meta-analysis again after excluding this study, and the results showed no effect on calibration, but reduced discrimination (Figure S4).

A


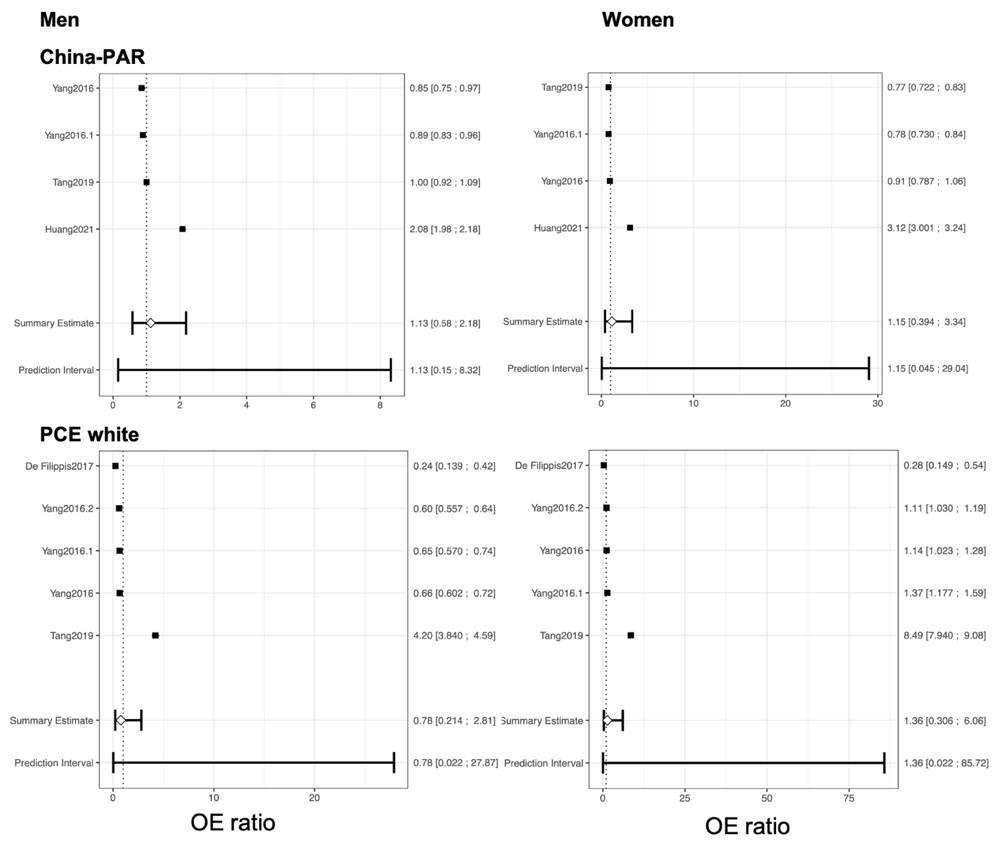


B


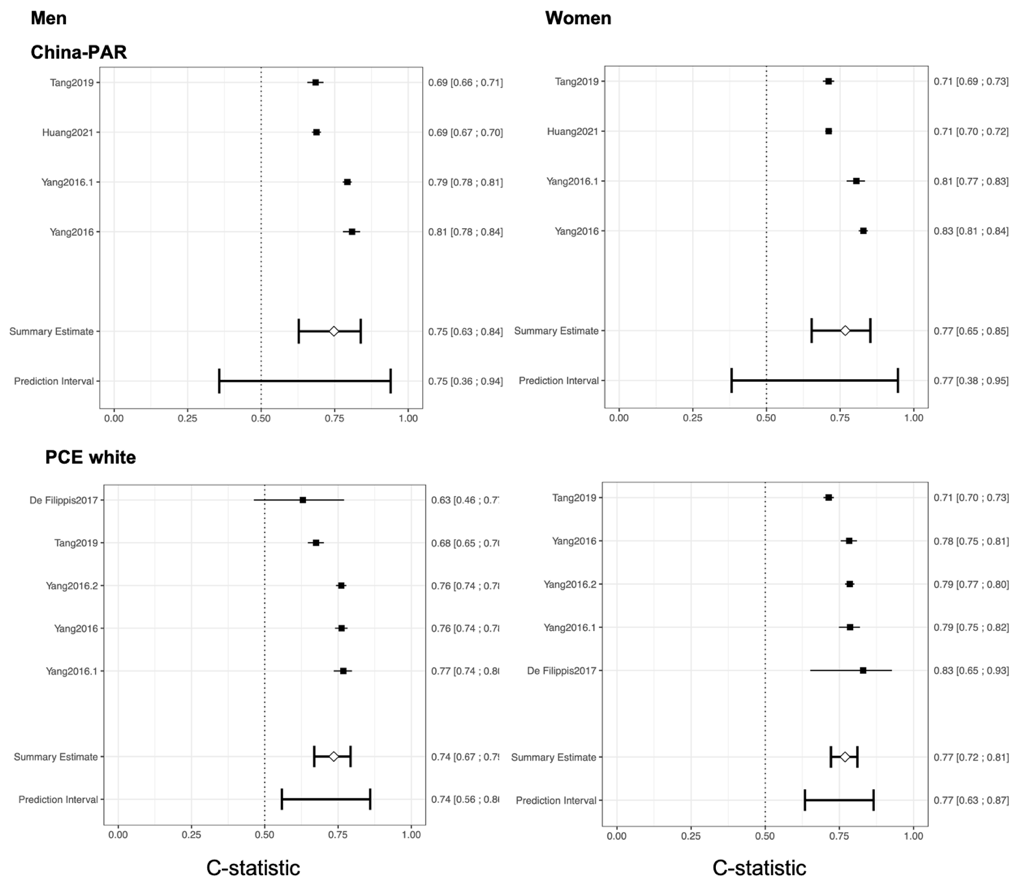


Figure S1. Sensitivity analyses considering risk of bias (A and B). China-PAR validations excluded studies by Jiang 2020 and Zeng 2008; PCE white validations excluded studies by Jiang 2020 and Lee 2015. PCE: Pooled Cohorts Equations, OE: observed expected.

A


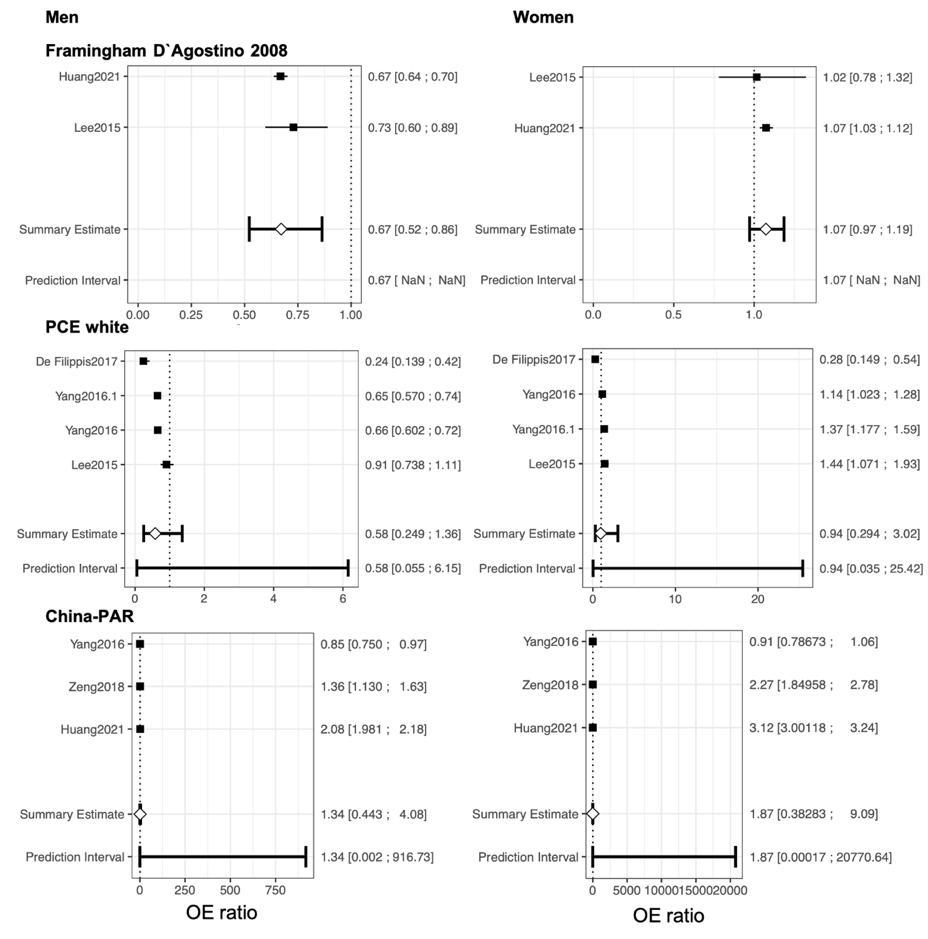


B


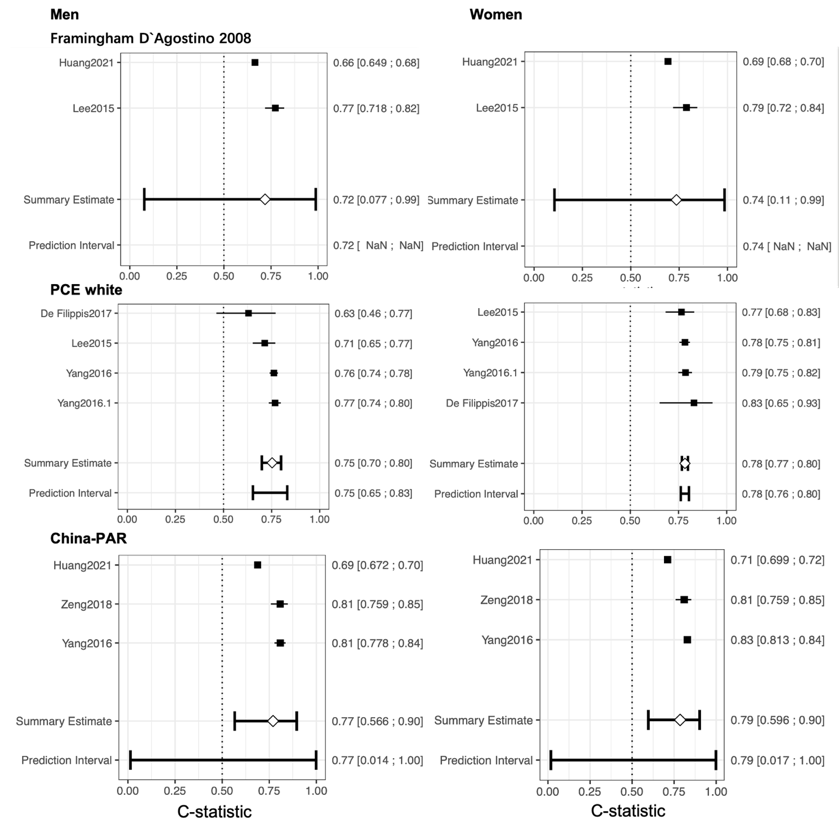


Figure S2. Sensitivity analyses considering less than 10-year prediction horizons (A and B). China-PAR and PEC validations both excluded studies by Yang 2016(CIMIC cohort), Jiang 2020 and Tang 2019; Framingham D`Agostino model validations excluded studies by Jiang 2020. PCE: Pooled Cohorts Equations, OE: observed expected.

A


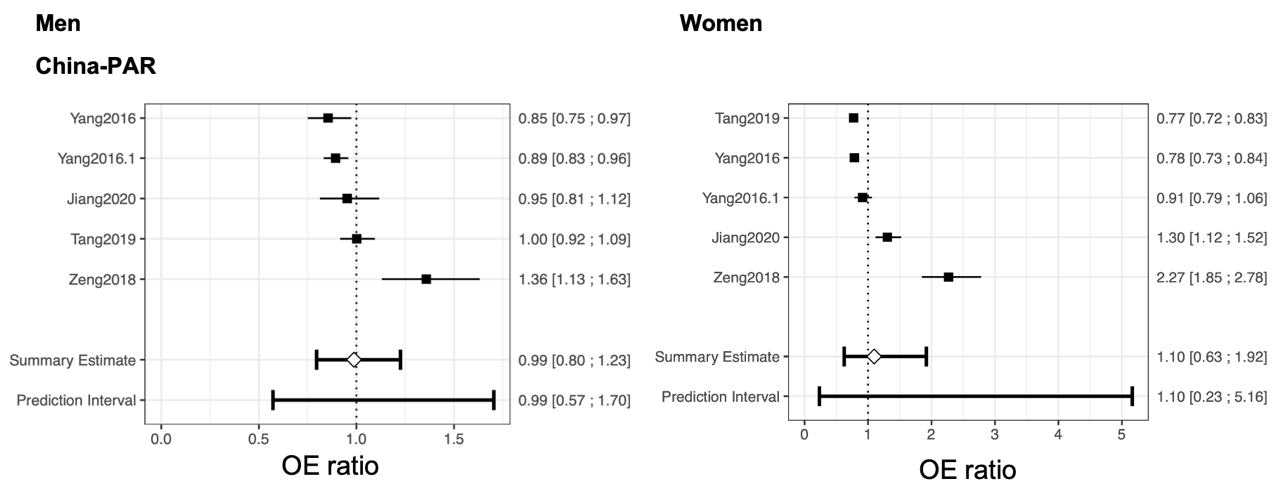


B


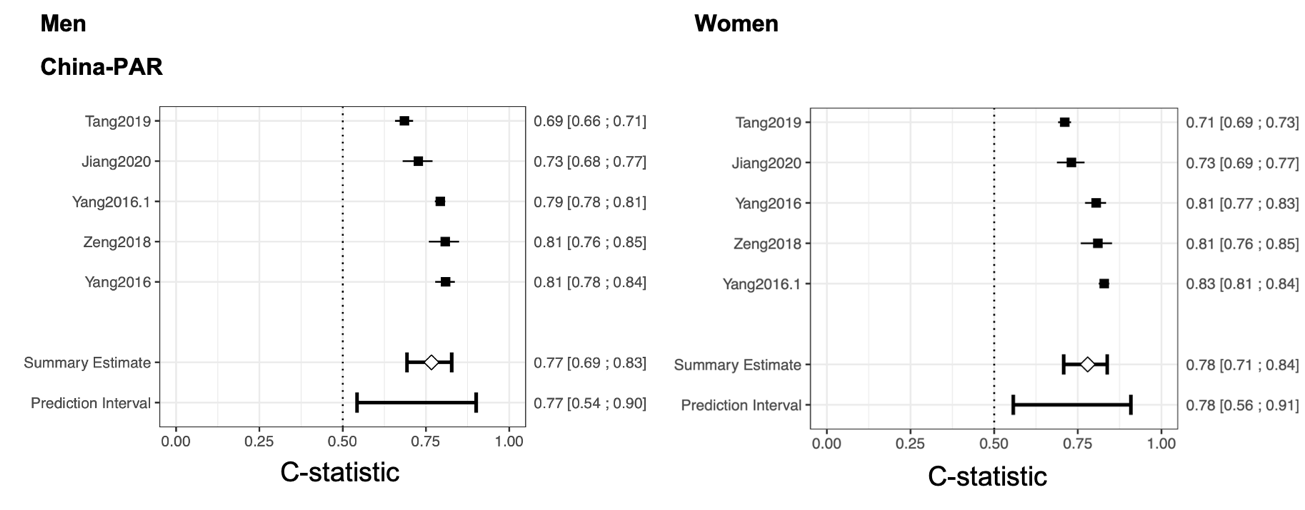


Figure S3. Sensitivity analyses considering age group for China-PAR model (A and B). OE: observed expected.

A


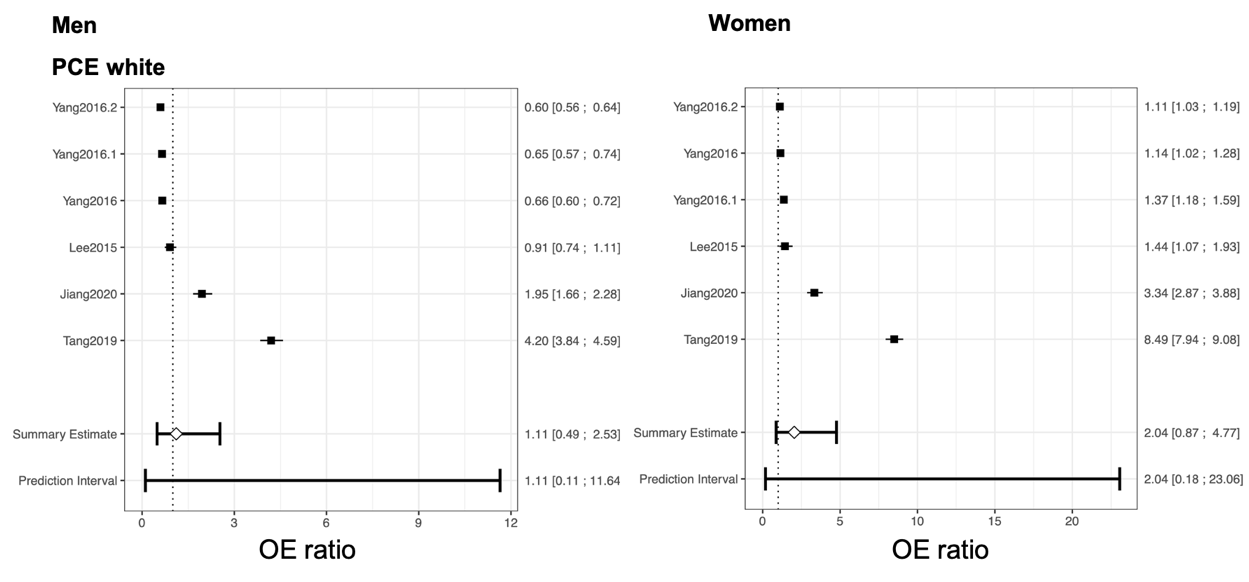


B


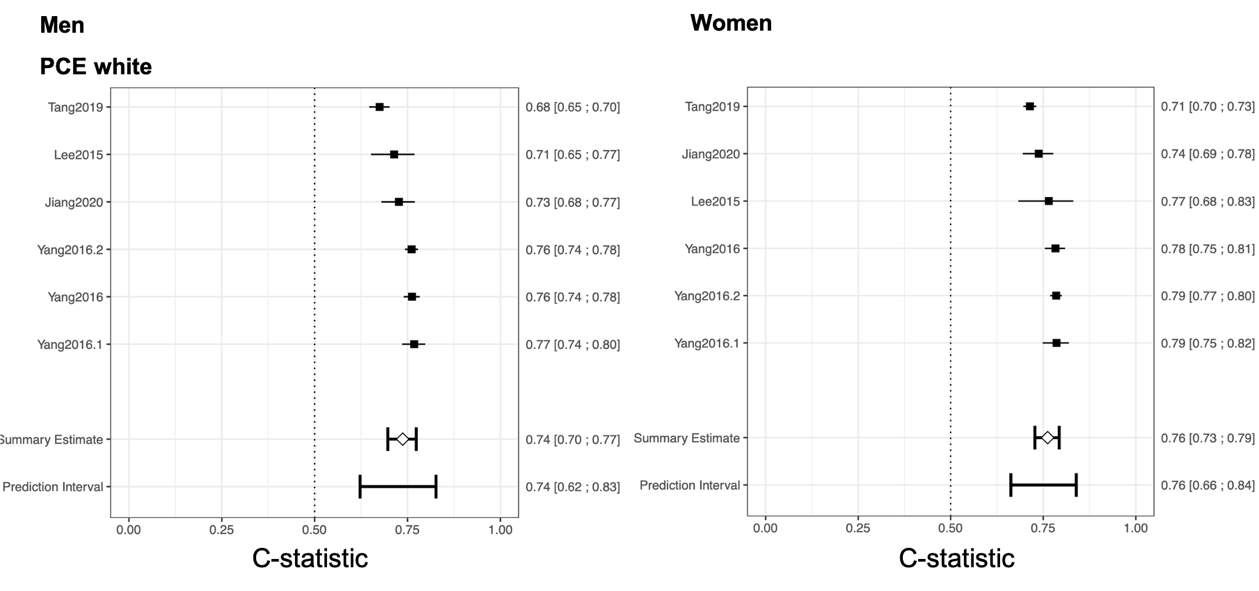


Figure S4. Sensitivity analyses considering ethic group for PCE model. PCE: Pooled Cohorts Equations, OE: observed expected.
